# Mandibular anterior crowding: normal or pathological?

**DOI:** 10.1590/2177-6709.23.2.030-036.oin

**Published:** 2018

**Authors:** Alberto Consolaro, Mauricio de Almeida Cardoso

**Affiliations:** 1 Universidade de São Paulo, Faculdade de Odontologia de Bauru (Bauru/SP, Brazil).; 2 Universidade de São Paulo, Faculdade de Odontologia de Ribeirão Preto, Programa de Pós-graduação em Odontopediatria (Ribeirão Preto/SP, Brazil).; 3 Faculdade de Medicina e Odontologia São Leopoldo Mandic, Programa de Pós-graduação em Ortodontia (Campinas/SP, Brazil.)

**Keywords:** Dental crowding, Orthodontic retainer, Dental resorption, Non-erupted teeth, Tensegrity, Dental concrescence.

## Abstract

The teeth become very close to each other when they are crowded, but their structures remain individualized and, in this situation, the role of the epithelial rests of Malassez is fundamental to release the EGF. The concept of tensegrity is fundamental to understand the responses of tissues submitted to forces in body movements, including teeth and their stability in this process. The factors of tooth position stability in the arch - or dental tensegrity - should be considered when one plans and perform an orthodontic treatment. The direct causes of the mandibular anterior crowding are decisive to decide about the correct retainer indication: Should they be applied and indicated throughout life? Should they really be permanently used for lifetime? These aspects of the mandibular anterior crowding and their implication at the orthodontic practice will be discussed here to induct reflections and insights for new researches, as well as advances in knowledge and technology on this subject.

## RESUMO

Os dentes ficam muito próximos quando estão apinhados, mas suas estruturas permanecem individualizadas e, nessa situação, o papel dos restos epiteliais de Malassez é fundamental para liberar o EGF. A tensigridade é um conceito chave para compreender as respostas dos tecidos submetidos às forças nos movimentos corporais, incluindo os dentes e sua estabilidade nesse processo. Os fatores da estabilidade de posição de um dente na arcada dentária — ou tensigridade dentária — devem ser considerados quando se planeja e finaliza um caso na prática clínica ortodôntica. As causas diretas do apinhamento dentário anteroinferior são determinantes para se refletir se a contenção deve ser mesmo indicada e aplicada por toda a vida e se, necessariamente, deve ser usada de forma permanente. Esses aspectos do apinhamento dentário anteroinferior e suas implicações na prática clínica serão aqui abordados para induzir reflexões e insights de novas pesquisas, bem como avanços no conhecimento e tecnologia sobre esse assunto.

The word crowding is originated from the verb to crowd, which means: 1. to press closely together; force into a confined space; cram; 2. to push; shove, 3. to fill to excess; fill by pressing or thronging into; 4. to place under pressure or stress by constant solicitation; 5. to gather in large numbers; throng; swarm; 6. to press forward; advance by pushing. 

The present paper focussed on the mandibular anterior crowding in the permanent dentition. The crowding can be classified as primary, secondary or tertiary, when it affects the mixed dentition in the first transitional period, second transitional period and permanent dentition, respectively.^6^ ^,^ ^7^ ^,^ ^8^

**Figure 1:**
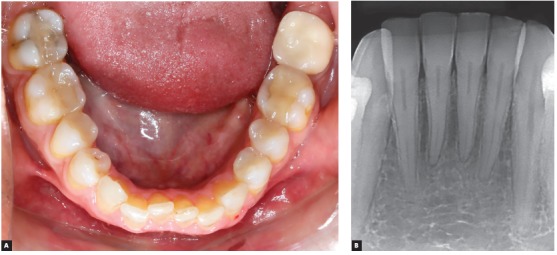
Mandibular anterior crowding, highlighting the proximity of the teeth (roots), maintaining their structures, without inflammatory resorption, dental ankylosis, nor concrescence.

Crowding represents one of the most frequent (perhaps the main) complaints of patients seeking orthodontists (Fig 1). Many questions about the mandibular anterior crowding tend to generate incomplete, evasive and reticent answers, since there are still many doubts about it, although it is a very studied subject in the literature.

Thus, the objectives of this paper are:

A) To answer the following questions:
  1^st^. Why do teeth become so close to each other, move from their original position in the arch, but do not contact each other, even on severe cases of crowding (Fig 1)?
  2^nd^. How are teeth so close, almost in contact, but with no alveolodental ankylosis, nor inflammatory resorptions?
  3^rd^. Why do not even very close teeth, like crowded teeth, evolve to concrescence?
B) To discuss the causes of the mandibular anterior crowding, with its implications in orthodontics practice, in a more inquiring way, in order to lead to reflections and insights. For this, we selected a few questions to guide this text, such as:
  1^st^. Is mandibular anterior crowding normal or pathological?
  2^nd^. Every patient, throughout life, will have mandibular anterior crowding, as suggested by the vast majority of orthodontists and the classical orthodontic books?
  3^rd^. What are the causes that directly act on the mandibular anterior crowding?

## TEETH ARE CLOSE AND CROWDED, BUT THE STRUCTURES REMAIN INDIVIDUALIZED

Even in the most severe crowding cases, the teeth do not touch one another. Their mineralized structures do not collide.^2^ And even if a root gets too close to other, neither inflammatory resorption nor ankylosis occurs, much less dental concrescence. Why does not this occur?

The periodontal space in which the periodontal ligament is located is maintained thanks to the constant liberation of EGF (Epidermal Growth Factor) by the epithelial rests of Malassez. This peptide is released by this epithelial web - spatially configured as a “basketball net” around the root - permeating between the fibers and the cells, “moistening” the periodontal ligament. 

In the periodontal ligament, the EGF has the function of stimulating the bone resorption at the periodontal face of the alveolus, if the deposition of new layers of fasciculate bone gets close to the tooth. In this way, the periodontal space is always maintained with 0.2 to 0.4-mm thickness.^1^

The osteoblasts have EGF receptors, but the cementoblasts, do not. Thus, resorption of the hard tissues occurs only on the bone surface of the periodontal ligament and is not visualized on its cementum surface. That’s how we explain why the alveolodental ankylosis does not occur with aging, even decades after a strict proximity between the alveolar bone and tooth root. 

Likewise, in cases of crowded teeth dangerously close to each other, there is no root resorption neither concrescence. Maintenance of the periodontal ligament also protects the root against inflammatory resorption, thanks to the epithelial rests of Malassez.^1^ ^,^ ^2^

The dental concrescence corresponds to the union of mature and erupted teeth by cement, which is very rare. In crowded teeth, this hardly happens, because as one tooth approaches another, alveolar bone remodeling is directed to where the occlusal forces, neighboring teeth and soft tissues are moving the tooth. In this bone remodeling, the periodontal space remains stable, thanks to the EGF that is continuously liberated by the epithelial rests of Malassez. As new layers of cement slowly build up, the periodontal thickness is maintained.

## TENSEGRITY CONCEPT IS THE KEY TO UNDERSTAND TISSUES ANSWERS

In animal and vegetal bodies, as well in objects, the supporting systems tend to receive and create forces in their structures, but, in the end, the forces cancel each other, with a resultant equal to zero. At the end of the action of these forces, with internal or external origin, and resultant equal to zero, the object or the anatomic structure will remain as it was originally. This indicates a full and perfect system of forces distribution - a balance -, whose property is called tensegrity. 

It is the same to a viaduct, a palace or to the head and the members of the human body, for example, but even on very simple things, as a decorative clay pot and the teeth positioning of the teeth steadily in the dental arch, by the linked action of tongue, lips, occlusion forces, neighboring teeth and bone dynamism. 

Any structure that returns to its original format after each applied force is in structural and functional balance. When this same structure gets modified in its shape by forces, we can have a new design, provided a new balance of forces is established, that is, we get a new tensegrity. If we do not reach a new tensegrity after all this change, is natural that everything returns to the original shape, to the normal balance, as if it were a recurrence to the originality.

Every time that the tensegrity of a cell with its own cytoskeleton is broken, or from a group of muscles and tendons and/or interrelated bones, the cells components will release many mediators, so that everything returns to its original shape. For example, the bones will be reabsorbed or undergo neoformation, vases will get larger, muscles will be sore and tense, everything to have tensegrity again, since the new shape gives back the tensegrity and equilibrates the system. 

Tensegrity represents the balance of a system of forces, in which they cancel each other and the resultant will be zero. The object or organ will thus remain in a stable shape, just like the bone as well. 

The concept of tensegrity was established by Richard Buckminster Fuller (1895-1983),^5^ an American genius who is considered a designer, an architect and a visionary writer. His main creation was the “geodesic dome”, which, with its triangular shapes, cables and balance, brings a very light sphere to protect what is inside it, as weapons, radars, machines and other activities protected from the weather, even in inhospitable environments, as deserts and wild valleys, and helped the American government to hide its radars and missiles. 

One of Fuller’s students was Kenneth Duane Snelson (1927-2016),^5^ an American sculptor and photographer who created sculptures and artworks based on tubes and cables, generating forms and structures almost suspended in the air. In many cases, they were simulations of molecules and microscopic structures. Snelson brought his work to the extreme of the tensegrity concept, almost taking for himself the concept of balance and force distribution, spreading it all over the world. The subject “tensegrity” was treated very elegantly by Donald E. Ingber in the context of life architecture, in an article published in the Scientific American^5^ on January of 1998. 

## STABILITY FACTORS OF A TOOTH POSITION IN THE DENTAL ARCH: THE DENTAL TENSEGRITY

Tensegrity is the term used to describe the concept of balance or stability of any system of forces of nature or made by humans, including the dental arch. When a system receives external or internal forces and the final resultant is equal to zero, it means that there is a balance in this system, that is, it has tensegrity, which can be analyzed exclusively in one tooth, one group of teeth, in the dental arch or across the face.

The systems of forces are dynamic and the external influences tend to modify them, but almost always temporarily or fleetingly, when they present tensegrity. The teeth on a dental arch should be on tensegrity offered by:

a) The teeth, in the interproximal surfaces.
b) Antagonists teeth in stable occlusion.
c) Forces generated in the functions of the tongue and other soft tissues, on the lingual side.
d) Cheeks, lips and other soft tissues, on the buccal side.
e) The adaptive and functional dynamism of bone tissue, constantly remodeling, guided by the dissipation of forces. This process generates forces that tends to take the tooth to other position, but the occlusion and all the other forces keep the tooth on the same position. The countless mitosis, cellular movements and the constant deposition of tissue matrix generate forces that add up and increase, while making opposition to other forces from other areas. The result, or the resulting forces are known as vectors - in this case, more specifically, as bone growth vectors. Even after the growth, with the maturation, these vectors keep existing with less intensity and frequency, with a much more adaptive nature. In the mandible, this growth is called later mandibular growth, or residual mandibular growth.
f) Growth vectors originated by functional and aesthetical adaptations derived from aging, which results in atrophy, hypertrophy or tissue hyperplasia, as the vertical dimension reduction caused by attrition, loss of muscles strength, reduction of soft tissue consistency and reduction of masticatory strength. This process generates forces that tend to bring the tooth to other position, but the occlusion and the other forces keep the tooth at the same position.

When one of these factors decreases, increases or changes, the balance, or tensegrity, is broken, and the tendency of the tooth is to slowly and gradually change its position, spinning within the alveolus, migrating one of its faces to the other side of the dental arch, which means that the teeth can become crowded. 

The dental arches are one of the parts of the organism under constant movements and loads during their functions. As the other members, the dental arches are in constant remodeling and functional adaptation.^3^ ^,^ ^4^ In a situation of balance or tensegrity, its shape and function remain normal. But the system may be modified, losing its tensegrity, until a new shape and balance position is obtained, which may not be esthetic and functionally convenient, such as a crowded dental arch in the anterior region. 

## THE DIRECT CAUSES OF MANDIBULAR ANTERIOR CROWDING

If one of the six determining factors of the dental tensegrity in the dental arch fails or reduces its effectiveness on stabilization, we may have dental crowding (from light to severe).

The mandibular anterior crowding in adults can be explained as a result of the change in the distance between the lower canines, although some retainers bonded to the canines may, eventually, be seen with crowding of the incisors. What could be the reasons for this intercanine width reduction at the lower arch? It could be due to a facial aging? Who changes first and is the cause of the other: the intercanine width or the mandibular anterior crowding?

We can probably find answers for a “natural” tendency to the mandibular anterior crowding in the following observations:

1. The mandible, even after the growth of the entire body has ceased, keeps growing, especially due to the condyle and its cartilaginous, bony and fibrous constitution. This would be a residual mandibular growth that would lead to a bone increase to the anterior region, with a subtle but efficient displacement of the teeth toward the midline.
2. Chewing promotes occlusal wear, compensated at the tooth by the continuous deposition of apical cementum, in a process known as passive and continuous dental eruption. Even preserving the height of the clinical crown, this process promotes changes at the occlusion, which needs to be compensated.
3. Interproximal contact points gradually turns into contact facets between the teeth, reducing the mesiodistal width between them as well as the total dental arch perimeter.
4. These changes micrometrically alter teeth position, and its tensegrity is recomposed from the bone remodeling and the terminal mandibular growth.

The need to keep the contact between teeth in the dental arch, reducing its perimeter, associated to the mandibular residual growth, clinically gives the impression that the teeth were “pushed” to the midline. The quest for tensegrity causes remodeling and mandibular growth to reposition teeth in the dental arch, constantly and at minimal levels, but on a daily basis. Any fail on the tensegrity factors can lead to the mandibular anterior crowding.

Even with enough space in the dental arch, can crowding occur? Maybe just arranging and earning space will not solve crowding. It would be necessary to orthodontically correct crowding, aligning teeth and reestablishing the tensegrity in a new reality, with well positioned mandibular teeth and in a good relationship with the maxillary ones and soft tissues.

Crowding should be considered a loss of tensegrity of teeth in the dental arch, and not simply a consequence of the lack of space. It should be identified which of the stability factors, or tensegrity, in the dental arch are failing or absent. 

The fact that mandibular anterior crowding is considered “natural” does not qualify it as “normal”, although this could lead to many controversial opinions. As an analogy, for comparative purposes, we could use the alopecia, or baldness, in the men, which happens with aging: it is natural, but can it be considered normal? 

## FINAL CONSIDERATIONS: SHOULD WE USE RETAINERS OR NOT? TEMPORARILY OR PERMANENTLY?

If, after orthodontic treatment, retainers use is mandatory, we must consider that:

1. The system is not in full tensegrity, in the long term. The teeth that were taken to the crowding did not finish “locked” by occlusion and other stability factors. There are still some forces acting in search of a tensegrity that was not obtained or was lost.
2. In the day to day of bone remodeling and reformatting, as well as in the physiology of the stomatognathic system, forces are generated that can easily break the obtained dental, bone and facial tensegrity. The factors of dental stability are not fully working in space and time.
3. The mandibular anterior crowding should be considered “normal” in the human being and it is inevitable during the aging process. The term normal is almost synonymous of “physiological”.

Considering our evolutionary stage, we should recognize that recommending the continued use of retainers in Orthodontics, with all the resulting discomfort, reflects the need to know more deeply the problem, allowing us to advance, from the technological point of view, in the purposes of resolution. 

How many researches we still need to make to dismiss the use of the retainers in the orthodontic practice? We’ll get there? Before that, we need to:

1. Recognize that the mandibular anterior crowding is a result of tensegrity loss in the dental arch.
2. Accept that tensegrity obtained at the beginning was not stable, permanent and definitive.
3. Understand and explain to the patients that the dental stability factors, or the dental tensegrity, may be modified by external factors, including facial aging.

And the cases of people who naturally, during life time, do not show mandibular anterior crowding? What are the factors that lead them to this situation? Is the intercanine width decrease that leads to the mandibular anterior crowding during aging, or this crowding is the factor that causes the decrease of the intercanine width?

This matter is not totally clarified in the literature, but a meticulous study of its evolution in time suggests that the mandibular anterior crowding represents the break of tensegrity in the dental arch. 

It is very likely that aging will only offer more opportunities in time - increasing the probability - for the causes that promotes loss of tensegrity in the dental arch to act, thus promoting the crowding. Let’s make more researches.
